# A class I odorant receptor enhancer shares a functional motif with class II enhancers

**DOI:** 10.1038/s41598-020-79980-x

**Published:** 2021-01-12

**Authors:** Tetsuo Iwata, Satoshi Tomeoka, Junji Hirota

**Affiliations:** 1 Center for Biological Resources and Informatics, Tokyo Institute of Technology, Yokohama, 226-8501 Japan; 2 Biomaterial Analysis Division, Technical Department, Tokyo Institute of Technology, Yokohama, 226-8501 Japan; 3 Department of Life Science and Technology, Graduate School of Life Science and Technology, Tokyo Institute of Technology, Yokohama, 226-8501 Japan

**Keywords:** Olfactory receptors, Transcriptional regulatory elements

## Abstract

In the mouse, 129 functional class I odorant receptor (OR) genes reside in a ~ 3 megabase huge gene cluster on chromosome 7. The J element, a long-range *cis*-regulatory element governs the singular expression of class I OR genes by exerting its effect over the whole cluster. To elucidate the molecular mechanisms underlying class I-specific enhancer activity of the J element, we analyzed the J element sequence to determine the functional region and essential motif. The 430-bp core J element, that is highly conserved in mammalian species from the platypus to humans, contains a class I-specific conserved motif of AAACTTTTC, multiple homeodomain sites, and a neighboring O/E-like site, as in class II OR-enhancers. A series of transgenic reporter assays demonstrated that the class I-specific motif is not essential, but the 330-bp core J-H/O containing the homeodomain and O/E-like sites is necessary and sufficient for class I-specific enhancer activity. Further motif analysis revealed that one of homeodomain sequence is the Greek Islands composite motif of the adjacent homeodomain and O/E-like sequences, and mutations in the composite motif abolished or severely reduced class I-enhancer activity. Our results demonstrate that class I and class II enhancers share a functional motif for their enhancer activity.

## Introduction

In the main olfactory epithelium (MOE), olfactory sensory neurons (OSNs) detect chemical stimuli in the external environment by expressing odorant receptor (OR) genes^1^. ORs, G protein-coupled receptors with a putative seven-transmembrane structure, evolved to adapt to species-specific chemical environments, resulting in the establishment and diversification of the largest gene family in vertebrate genomes^2^. Mammalian OR genes are classified into two classes, class I and class II, based on the homology of their deduced amino acid sequences^3^. Class I ORs resemble the OR family first identified in fish and frogs^4,5^, whereas class II ORs are specific to terrestrial animals^6^. It has been presumed that class I ORs detect hydrophilic odorants and class II ORs detect hydrophobic odorants^7^.

Class I and class II genes have different genomic organization^8,9^. There are 129 functional class I genes in the mouse genome, all of which are embedded in a single huge cluster within a ~ 3 Mb genomic region on chromosome 7, forming one of the largest gene clusters in the genome. In contrast, the ~ 950 class II genes are distributed throughout almost all chromosomes. Each olfactory sensory neuron expresses a single functional allele of a single OR gene from the repertoire of class I or class II ORs^10–14^. This one neuron-one receptor rule is established during OSN differentiation by the two sequential steps of OR class choice and the expression of a single OR gene from the corresponding class of OR repertoires.

The OR class choice is regulated by the zinc finger transcription factor Bcl11b (also known as Ctip2), which functions as a binary switch to select OR class^14^. In the absence of Bcl11b, the class I gene is selected by default, whereas class I gene expression is suppressed in the presence of Bcl11b, leading to selection of the class II gene. The singular OR gene expression can be further divided into two processes, the selection of a single OR allele and the subsequent maintenance of transcription of that allele. For the selection of a single OR allele choice, Magklara et al*.* demonstrated that all OR genes are epigenetically silenced prior to OR selection, and that a single OR allele escapes stochastically from heterochromatic silencing^15^. Subsequently, the expressed OR protein elicits a negative-feedback signal to prevent the activation of additional OR genes^16,17^.

In addition to epigenetic regulation, it has been demonstrated that *cis*-regulatory elements/enhancers are involved in the transcriptional activation of a single OR allele^13,16,18–20^. OR enhancers activate a single OR allele within the linked cluster. A few cis-regulatory elements have been identified experimentally in mouse class II genes from ~ 60 candidate elements. The H, P, and Lipsi elements control 7–10 class II genes of the linked clusters within a ~ 200 kb genomic range ^16,18–21^. Together with the *cis*-regulatory effect, *trans*-interaction among the class II enhancers (Greek islands) has been demonstrated to play an important role in the formation of OR compartment and interchromosomal enhancer hub to express a single OR gene, in which intergenic class II enhancers (Greek islands) form a super-enhancer associating with a single active OR gene^22^. Recently, the J element, a *cis*-regulatory element of the mouse class I genes was identified, which exhibits unique features with respect to its extraordinary long-range regulation and is evolutionarily conserved in mammalian species^13^. The deletion of the J element was shown to result in a significant decrease in the mRNA levels of 75 class I genes over the whole 3 Mb cluster. Intriguingly, the J element regulates class I gene expression of a much larger number of genes and over a greater genomic distance than not only class II enhancers (comparing the *cis*-effect) but also any other known enhancer elements, for example, the largest number of genes has been found to regulate is ~ 30, in the cluster control region of the protocadherin-β and γ-genes^23^, and the longest genomic distance across which has been found to act is ~ 1.3 Mb, for the 3′ enhancer of the protooncogene *Myc*^24^.

In the class II enhancers, the H and P elements contain conserved sequence motifs of multiple homeodomain sites and neighboring O/E-like sites^18^. Mutation analysis of the core H element demonstrated that these conserved motifs are essential for enhancer activity. This motif organization of homeodomain and O/E-like sites was also found in the 430-bp core J element, which is conserved in mammalian species from the platypus to the humans^13^. Recently, deletion of the 912-bp region containing the core J element by genome editing was shown to result in a massive decrease in class I gene expression, suggesting that the core J element plays an important role in class I gene expression^25^. Interestingly, the core J element contains a class I-specific novel conserved motif (5′-AAACTTTTC-3′). In this study, to elucidate the molecular mechanisms underlying the class I-specific enhancer activity of the J element, we analyzed the core J sequence to identify the essential region and motifs for class I OSN-specific enhancer activity.

## Results

### The class I-specific motif is not essential for the enhancer activity

**Figure 1:**
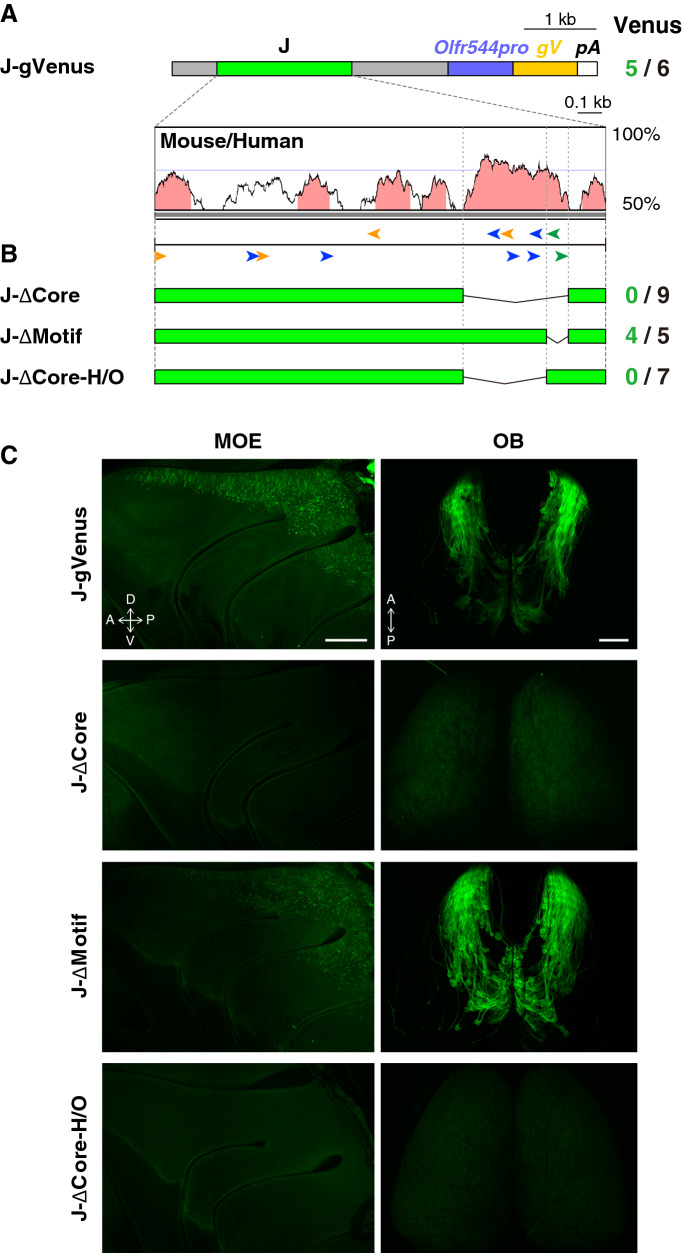
The 330-bp core J-H/O region, but not the class I-specific conserved motif, is necessary for the enhancer activity of the J element. (**A**) The J-gVenus transgene construct and schematic representation of putative binding motifs of the homeodomain site (blue arrowheads), O/E-like sites (orange arrowheads), and class I-specific conserved motif, the AAACTTTTC site (green arrowheads), in the J element, together with the VISTA plot between mouse and human. *Olfr544pro*, promoter region of *Olfr544*; *gV*, membrane-targeted *Venus* reporter (*gapVenus*); *pA*, polyadenylation sequence. The number of reporter-positive independent founders and lines among the total analyzed is shown on the right^13^. (**B**) Deletion series of the J-gVenus transgene. The number of gapVenus-positive independent founders out of the total number analyzed is shown on the right. (**C**) Whole-mount fluorescent images of the turbinate of MOE and the dorsal OB of J-gVenus, J-ΔCore, J-ΔMotif, and J-ΔCore-H/O Tg founder mice. The scale bar is 500 μm.

We previously demonstrated that the J-gVenus transgene, in which the 3.8-kb NcoI fragment including the J element was placed upstream of the 0.9-kb *Olfr544* promoter region and the *gapVenus* reporter gene, exhibited reproducible and robust expression of gapVenus specifically in class I OSNs (Fig. 1A)^13^. Because the *Olfr544* promoter region itself could not activate reporter gene expression^13,26^, and was replaceable by the *SV40* minimal promoter for the class I-specific gene expression of the J element (Fig. 1), we concluded that the J element is responsible for expression of the transgene. Within the J element, the 430-bp region (the core J element) that corresponds to the highest homology region between the mouse and human J elements contains the novel conserved motif of AAACTTTTC in addition to the cluster of multiple homeodomain and neighboring O/E-like sites, as in the class II enhancers^13^(Fig. 1A). To identify the minimum requirement for the enhancer activity of the J element and function of the conserved motifs, we constructed a deletion series of transgenes based on the J-gVenus construct, and generated transgenic mice (Fig. 1B).

First, we constructed the J-ΔCore transgene by deleting the core J sequence from the J-gVenus transgene and generated transgenic mice using the *Tol2* system. None of the nine founders carrying the J-ΔCore transgene showed gapVenus expression, whereas five out of six founders/lines of the J-gVenus Tg mice demonstrated the class I OSN-specific gene expression pattern, indicating that the core J is essential for the enhancer activity of the J element (Fig. 1B,C). This result is comparable to the result of deletion of the 912-bp region including the core J element in vivo in a previous study^25^.

As the AAACTTTTC motif is conserved through mammalian genomes from the platypus to humans and is specific to the class I enhancer^13^, it is possible that this motif is responsible for class I OSN-specific enhancer activity. To test the function of the class I-specific conserved motif, we deleted a region containing the class I-specific motif to retain the homeodomain and O/E-like sites to generate the J-ΔMotif transgene. Four out of five founders exhibited gapVenus fluorescence in both the MOE and olfactory bulb (OB) similar to the J-gVenus Tg mice, indicating that the novel conserved motif of AAACTTTTC specific to the class I enhancer is not essential for the enhancer activity of the J element (Fig. 1B,C).

### The 330-bp core J-H/O is necessary and sufficient for class I-specific enhancer activity

The results of the J-ΔMotif transgene reporter assay together with that of the J-ΔCore transgene suggested that the remaining 330-bp region containing the homeodomain and O/E-like sites designated as the core J-H/O is important for the enhancer activity of the J element. To test this, we deleted this region and generated transgenic mice carrying the J-ΔCore-H/O transgene. None of the seven founders of the J-ΔCore-H/O Tg mouse line exhibited gapVenus fluorescence, indicating that the 330-bp core J-H/O is necessary for the class I-specific enhancer activity (Fig. 1B,C).

**Figure 2:**
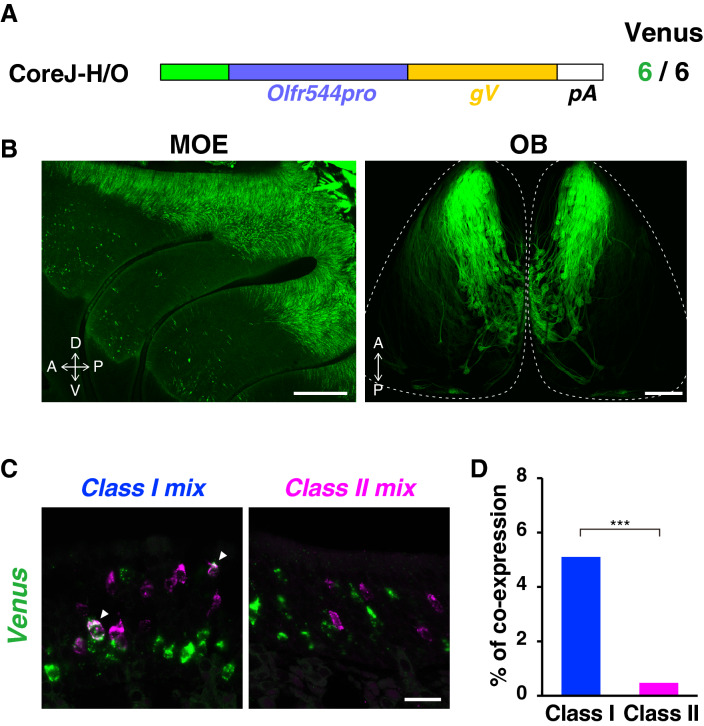
The 330-bp core J-H/O is sufficient for the class I OSN-specific enhancer activity. (**A**) Schematic representation of the CoreJ-H/O transgene construct. The number of gapVenus-positive independent founders out of the total number analyzed is shown on the right. (**B**) Whole-mount fluorescent images of the turbinate of MOE and the dorsal OB of CoreJ-H/O Tg F1 mice (Line#10). The scale bar is 500 μm. (**C**) Confocal images of two color ISH of *Venus* (green) with mixed probes of class I or class II OR genes (magenta) in CoreJ-H/O Tg mice. Arrowheads indicate the cells co-expressing Venus and OR genes. The scale bar is 20 μm. (**D**) Bar graph showing the percentage of *Venus*-positive cells that were labeled with the class I or class II OR mixed probes (1058 cells for class I, 1057 cells for class II from three Tg mice). Co-expression preferentially occurred in class I OSNs. Fisher's exact test; *p* = 1.1 × 10^–11^, ***p < 0.001.

We further examined if the core J-H/O is sufficient for enhancer activity by constructing a CoreJ-H/O transgene, in which the 330-bp core J-H/O element was placed upstream of the *Olfr544* promoter region and the *gapVenus* reporter gene (Fig. 2A). All six founders of the CoreJ-H/O Tg line showed a similar class I OSN-specific gene expression pattern to that of the J-gVenus Tg mice in both the dorsal MOE and the dorsal OB (Fig. 2B). To confirm the class I OSN-specific enhancer activity, we analyzed the co-expression of gapVenus with class I or class II OR genes in CoreJ-H/O Tg mice by two-color ISH (Fig. 2C). Quantification analysis of 2115 OSNs from three independent mice showed that gapVenus-expressing OSNs predominantly co-labeled with class I genes but not with class II genes (class I, 54/1058, co-expression rate = 5.1%; class II, 5/1057, co-expression rate = 0.47%; Fisher's exact test, *p* = 1.1 × 10^–11^; Fig. 2D). Together, these results indicate that the 330-bp core J-H/O element is necessary and sufficient for class I OSN-specific enhancer activity.

### A conserved motif responsible for the class I-specific enhancer activity

**Figure 3:**
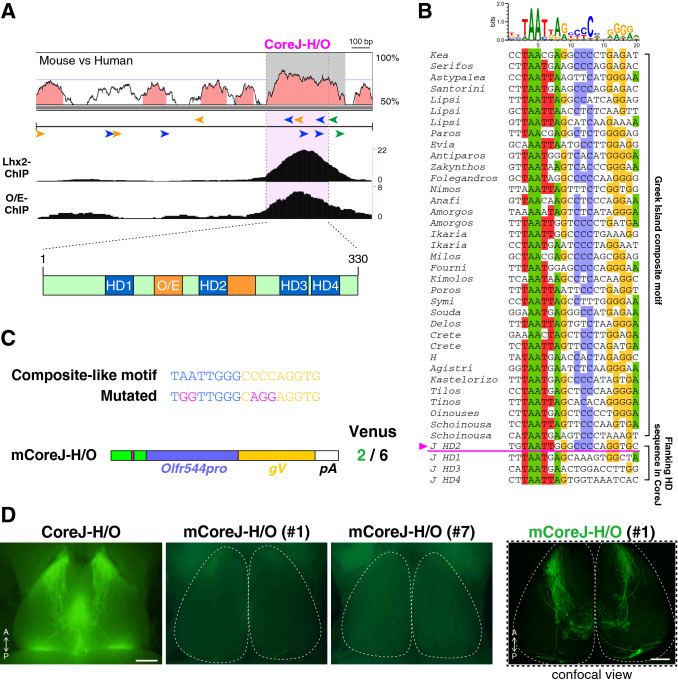
The composite motif in the core J element is essential for the enhancer activity. (**A**) ChIP-seq data of Lhx2 and O/E (Ebf) proteins. ChIP-seq peaks of Lhx2 and O/E are found in the core J-H/O sequence, suggesting that Lhx2 and O/E bind to the homeodomain and O/E-like sites in the core J-H/O element. The ChIP data were retrieved from GSE93570^30^. (**B**) Multiple alignment of the representative Greek Islands composite motifs in the class II enhancers and four homeodomain sites in the core J-H/O sequence (HD1–HD4 in (**A**). Comparison of the sequences revealed that the HD2 sequence belongs to the composite motif. A motif log shows all high-scored-composite motif sequence found in Greek island. Positions where matched nucleotides with at least 50% identity among the Greek island composite motifs are shaded by nucleotide identity: A = green, C = blue, G = yellow, T = red. (**C**) Schematic illustration of the mutations introduced into the composite motif and the mutated CoreJ-H/O transgene. The number of gapVenus-positive independent founders out of the total number analyzed is shown on the right. Note that two founders showed very weak fluorescent signals, which were hardly detected under the imaging conditions optimized to the CoreJ-H/O Tg mice. (**D**) Whole-mount fluorescent images of the dorsal OB of CoreJ-H/O and mCoreJ-H/O Tg mice. The left three panels are dorsal fluorescent images of the OBs of Core J-H/O (control) and mCoreJ-H/O (#1: very weak reporter expression; and #7: no reporter expression) Tg mice, taken under the same excitation and acquisition (gain and exposure time) conditions using a fluorescent stereomicroscope. The right panel is a confocal image to visualize the axonal projection domain of the founder #1, whose reporter expression was too weak to visualize in a fluorescent-stereomicroscopic image. Dotted lines indicate the OB outline. The scale bar is 500 μm.

Within the core J-H/O sequence, four homeodomain sites and one O/E-like site were evolutionarily conserved in mammals from the platypus to humans. In OSNs, the transcription factors Lhx2 and O/E family proteins (O/Es, Ebfs) bind to the homeodomain and O/E-like sites, respectively^27–30^. Thus, we first checked the binding of Lhx2 and O/E proteins to each motif sequence based on the ChIP-seq database for these transcription factors in mature OSNs, and found that both Lhx2 and O/E had peaks in the core J-H/O region^30^ (Fig. 3A). Recently, genome-wide searches for intergenic OR enhancers uncovered ~ 60 candidate enhancer elements (Greek Islands)^20,30^. Sequence analysis of the Greek Islands revealed a novel motif, the composite of adjacent homeodomain and O/E-like sequences, which play an important role in class II enhancer functions by recruiting Lhx2 and O/Es^30^. Interestingly, one of the four homeodomain sites in the core J-H/O region, HD2, was found to be the composite motif (Fig. 3B).

To examine whether this composite motif is important for the class I enhancer activity, we introduced point mutations into the motif by replacing AA with GG in the homeodomain sequence and CCC with AGG in the O/E-like sequence in the core J-H/O transgene to generate a mutated core J-H/O (mCoreJ-H/O) transgene (Fig. 3C). We obtained six founders of the mCoreJ-H/O Tg mouse line. Fluorescent signals of gapVenus were not detected in four founders and hardly detected in two founders. (Fig. 3D). These results indicate that the composite motif found in the core J-H/O plays a central role in the enhancer activity, and suggested that the remaining homeodomain and O/E-like sites may also contribute to the class I-specific enhancer activity.

## Discussion

In this study, we characterized the functional J element, and demonstrated that the 330-bp core J-H/O element is necessary and sufficient to drive class I OSN-specific gene expression. The length of the core J-H/O element is similar to that of the core H (187 bp) and P (317 bp) elements^12,18^. In addition, the motif organization of homeodomain and neighboring O/E-like sites is shared among them, suggesting that these features are common in class I and class II, and are important for their function as OR enhancers. Lhx2 and O/Es bind to the homeodomain and O/E-like sites, respectively, in the core J-H/O element as well as class II enhancer/promoters^27,29,30^, suggesting that Lhx2 and O/Es play critical roles in class I gene expression as well as in class II. However, while a knockout mutation in *Lhx2* precluded expression of class II genes, most class I genes were still expressed, though the mRNA levels and the number of expressing OSNs decreased, suggesting the existence of both Lhx2-dependent and Lhx2-independent mechanisms for class I gene expression. Indeed, it has been reported that not only Lhx2 but also Emx2 binds to the olfactory homeodomain sites, and targeted deletion of the *Emx2* gene altered the frequency of expression of OR genes, including class I^29,31^.

Motif analysis identified that one of the four homeodomain sites in the core J-H/O element (HD2 in Fig. 3) is the Greek Islands composite motif, a critical motif for some class II enhancer activities^30^. Our mutagenesis study demonstrated that the composite motif in the core J-H/O element is also important for enhancing class I OSN-specific gene expression as in the class II enhancers. However, the mutations in the composite motif did not abolish the enhancer activity completely; two out of the six founders exhibited very weak but specific enhancer activity in class I-OSNs, suggesting that the remaining three homeodomain sites (HD1, 3, and 4 in Fig. 3) and an O/E-like site may contribute to the enhancer activity of the J element. Because multiple homeodomain sites are frequently found in class II enhancers/promoters^20,30,32–34^, they may cooperate to regulate enhancer function. For example, HD1 (AAACTTTTAATGA) in the core J-H/O element is similar to the extended homeodomain sequence of AACTTTTTAATGA found in the H and P elements, and in the P3 promoter^35^. Although the extended homeodomain sequence is distinct from the composite motif, tandem repeats of the extended homeodomain sequence markedly increase transgene expression^35,36^. It is possible that the extended homeodomain sequence of HD1 cooperates to regulate the enhancer activity with the composite motif in HD2.

Because the class I-specific conserved motif of AAACTTTTC in the J element was perfectly conserved in mammalian species from the platypus to humans^13^, we expected a critical role in the class I-specific enhancer activity of the J element. Contrary to our expectations, however, a series of transgenic reporter assays showed that the class I-specific conserved motif is not required for class I OSN-specific transcriptional activation. What is the function of the class I-specific motif? The major difference between the J-element and class II enhancers is the scale of action. Class II enhancers regulate the expression of 7 to 10 genes within approximately 200 kb of genomic distance. In contrast, the J element regulates the expression of 75 genes over a genomic region of approximately 3 Mb. The 780-bp ZRS, a *cis*-regulatory element responsible for the spatiotemporal control of *sonic hedgehog* (*Shh*) at a distance of ~ 800 kb in the limb bud, is composed of two distinct domains^37^. Deletion of the 302-bp on the 3′ side of the ZRS in vivo abolished the expression of *Shh*. However, in Tg mice carrying the reporter transgene of the other part of the ZRS, that is, the 3′ side deletion of the ZRS, the endogenous expression pattern of the reporter gene is replicated. As in the ZRS, it is possible that the class I-specific motif is responsible for the ultra-long-range action of the J element across the entire cluster, and the composite motif, together with other homeodomain sites, plays an important role as an OR enhancer. This possibility will be examined by deleting the AAACTTTTC motifs from the J element by genome editing, which should reveal its function in vivo.

Overall, we identified that the core J-H/O element is necessary and sufficient for class I OSN-specific enhancer activity and the composite motif plays a central role in the enhancer activity as in the class II enhancers. Thus, the activation mechanism of each enhancer uses a common sequence motif, and the functional motif sequences of class I and class II enhancers do not by themselves define class specificity. What mechanisms determine class specificity? Recently, we demonstrated that Bcl11b, a zinc finger transcription factor determines the OR class to be expressed in mouse OSNs, and demonstrated that the OR class choice is established at the level of OR enhancer activation^14^. In the absence of Bcl11b, the class I enhancer is activated throughout the MOE, whereas the class II enhancer is suppressed. The class I enhancer activity is suppressed in the presence of Bcl11b, which in turn permits the activation of class II enhancers, resulting in the expression of class II genes. Because the depletion of Bcl11b, even after the terminal differentiation into neurons, can switch the enhancer activation from class II to class I in class II-characteristic OSNs, i.e., Acsm4 (also known as O-MACS) and NQO1-negative ventral OSNs, class specificity is determined by the absence (class I) or the presence (class II) of Bcl11b. One new question has been raised. How Bcl11b suppresses the J element? Because the class I-specific conserved motif of AAACTTTTC is inconsistent with GC-rich consensus sequence of Bcl11b binding^38,39^ and the alignment of the core J sequences in eleven mammalian species did not reveal any conserved motifs other than the lass I-specific conserved motif, the composite motif, and homeodomain and O/E-like motifs^13^, Bcl11b may suppress the J element indirectly. Because Bcl11b recruits the nucleosome remodeling deacetylase (NuRD) complex^40^, the suppressive effects of Bcll1b on the J element may involve epigenetic modifications and chromatin remodeling.

In summary, in this study we identified the functional J element, and through examination of the effects of deletion and point mutations, demonstrated that class I and class II enhancers share the functional motif for their enhancer activities.

## Methods

### Animals

All mice were housed under standard conditions with a 12 h light/dark cycle, and access to food and water ad libitum. Mutant and wild-type mice of both sexes at 4–6 weeks of age were used for the experiments. All mouse studies were approved by the Institutional Animal Experiment Committee of the Tokyo Institute of Technology and were performed in accordance with the institutional, governmental ARRIVE guidelines. J-gVenus transgenic (Tg) mice were generated as described previously^13^.

A deletion series of J-gVenus Tg mice, CoreJ-H/O, and mutated-CoreJ-H/O (mCoreJ-H/O) Tg mice were generated using the *Tol2* cytoplasmic microinjection method, which is suitable for the founder assays of Tg mice because of its high efficiency of transgene integrations into multiple integration sites (usually 6–8 integration sites/founder) and a single copy of transgene for each integration site^26,41^. DNA solution containing 20 ng per μL circular *Tol2*-transgene plasmid and 25 ng per μL transposase mRNA was injected into the cytoplasm of B6C3F1 mouse zygotes (Japan SLC, Inc.). To obtain B6C3F1 zygotes, B6C3F1 female mice over four weeks old were treated with superovulation, and then mated with adult B6C3F1 male mice. An Olympus IX-71 microscope equipped with a micromanipulator transgenic system (Narishige) and FemtoJet system (Eppendorf) was used for microinjection. Injected eggs were transferred to the oviducts of pseudopregnant female ICRs (over six weeks old, Japan SLC, Inc.). The founders were screened by PCR with the *Venus* primer set of 5′-GCAAGCTGACCCTGAAGCTG-3′ and 5′-TTGCTCAGGGCGGACTGGTA-3′^26,41^.

### Transgene construction

Transgenes of J-ΔCore, J-ΔMotif, and J-ΔCore-H/O were constructed based on the J-gVenus transgene^13^. PCR fragments were amplified using the 3.8 kb Nco I fragment of the J-gVenus transgene as a template and the following sets of primers: a common forward primer of 5′-GCTCATCTCGATGCAGATCTC-3′, three reverse primers of 5′-GAAGATCTTAAGTCCCCATTGATGCCC-3′ (for J-ΔCore), 5′-GCGAGCTCTAAGTCCCATTGATGCCC-3′ (for J-ΔMotif), 5′-GAAGATCTAGAGCGAGAAAAGTTTGAGCC-3′ (for J-ΔCore-H/O). The amplified fragments were ligated into the BglII-BglII site for J-ΔCore, the BglII-SacI site for J-ΔMotif, and the BglII-BglII site for the J-ΔCore-H/O of the 3.8 kb J fragment to generate each target deletion. The deletion series of the 3.8 kb J fragment was inserted into the reporter vector, a modified pBluescript II SK(+) vector containing the *gapVenus-pA* fragment and the PCR-amplified *Olfr544* promoter fragment (~ 870 bp upstream of TSS plus ~ 40 bp of noncoding exon). For the core J-H/O transgene, a core J fragment was amplified using the following primers of 5′-GCTCTAGAACTTAGTGTCCCTCGGGCTTG-3′ and 5′-CGCGTCGACTCACACACTAAAAATCACTCTATCTC-3′, and a SalI-SacI fragment of the amplified fragment was ligated into the above reporter vector. For the mCore J-H/O transgene, point mutations were introduced by inverse PCR using the following primers: 5′-CTCCTGCCCAACCACAGCAGCCTGGGCCTAA-3′ and 5′-TGTGGTTGGGCAGGAGGTGCCCTG CTGAGTCT-3′.

### Analysis of whole-mount specimens

Fluorescent images of gapVenus signals in whole-mount specimens were taken with an Olympus SZX10 fluorescent stereomicroscope with a DP71 digital CCD camera and a Leica SPE confocal microscope. Confocal images were collected as z-stacks and projected onto a single image for display. Images were adjusted and merged using Adobe Photoshop CC2018.

### Two-color in situ hybridization

Probes for *Olfr78*, *Olfr544, Olfr552, Olfr578*, *Olfr672*, *Olfr692, Olfr19*, *Olfr54*, *Olfr73*, *Olfr151*, *Olfr521*, *Olfr878*, and *Venus* (*EGFP*) were prepared as previously described^13^. Briefly, all riboprobes were synthesized by in vitro transcription using T3, T7, or Sp6 RNA polymerase (Roche, 11031163001; 10881767001; 10810274001) with hapten-labeled UTP of digoxigenin (DIG) (Roche, 11277073910) or fluorescein (FLU) (Roche, 11685619910) in the presence of an RNase inhibitor, RNasin (Promega, N2111). The following mixed probes were used for two-color in situ hybridization (ISH); class I mix: *Olfr78*, *Olfr544, Olfr552, Olfr578*, *Olfr672*, and *Olfr692*; and class II mix: *Olfr19*, *Olfr54*, *Olfr73*, *Olfr151*, *Olfr521*, and *Olfr878*.

Mice were transcardially perfused with 4% paraformaldehyde in PBS. Dissected MOE tissues were post-fixed overnight at 4 °C. The tissues were then decalcified in 0.45 M EDTA in PBS for at least 2 days. After cryoprotection with 15% and 30% sucrose in PBS, tissue samples were embedded in FSC 22 Frozen Section Media (Leica Biosystems, 3801481), and sectioned coronally at 12 μm thickness using a cryostat (Microm HM505E). Sections were collected on MAS-coated glass slides (Mastunami, S9441).

For two-color ISH, the post-fixed sections were pretreated according to the methods described previously, and hybridized with hapten-labeled probes at 65 °C for overnight. After stringent washing, FLU-labeled OR probes and a DIG-labeled EGFP probe were sequentially detected at room temperature according to the following procedures: the sections were treated with 1% (v/w) DIG blocking reagent (Roche, 11096176001) and an anti-FLU-biotin antibody (1/1000 dilution, Vector, BA-0601) in TBST buffer (100 mM Tris–HCl, pH 7.5; 150 mM NaCl with 0.01% Tween 20) for 60 min each. The sections were incubated with avidin–biotin complex (Vector, VECTASTAIN Elite ABC Kit, BA-1400) for 30 min, and then with biotin-tyramide plus solution (1/50 dilution in Plus amplification diluent, PerkinElmer, TSA Plus Biotin Kit, NEL749B001KT) for 10 min. To inactivate horse-radish peroxidase (HRP), sections were incubated in 3% H_2_O_2_/PBS for 60 min. Following the inactivation of HRP, the sections were incubated with anti-DIG-HRP antibody (1/1000 dilution, Roche 11207733910) in DIG-blocking solution for 60 min, with Cy3-tyramide plus solution (1/50 dilution in Plus amplification diluent, PerkinElmer, TSA Plus Cyanine 3 System, NEL744B001KT) for 10 min, followed by Streptavidin-Alexa488 (1/1000 dilution, Thermo Fisher, S11223) in TBST. Between each treatment, the sections were washed three times (10 min each) in TBST at room temperature. Fluoromount mounting medium (Diagnostic BioSystems, K024) was used for fluorescent detection. The fluorescent images were taken with a Leica SPE confocal microscope. All fluorescence images were optimized (brightness and contrast) using Adobe Photoshop CC2018 software.

### Motif analysis

Chromatin immunoprecipitation-sequencing (ChIP) data for Lhx2 and O/E proteins (Ebfs) were retrieved from GSE93570^30^. The representative composite motif sequences described in Fig. 3, Supplement Fig. 1A of Monahan et al*.* were compared with the homeodomain sequences in the core J element^30^. The motif logo was created by Weblogo v3.7 (http://weblogo.threeplusone.com/create.cgi) using all high scored-composite motif sequences from the Greek Islands^42^.

### Statistical analysis

Statistical analysis and graphical representation were performed using Microsoft Excel. No randomization method was used, and no statistical methods were used to predetermine the sample size. The sample sizes in this study were generally similar to those used by other studies in the field. Quantification of the number of OSNs co-labeled with OR mix probes and EGFP probe was done blinded to exclude experimenter bias.

## Supplementary Information

Supplementary Information.
