# Supplementary material for: A class I odorant receptor enhancer shares a functional motif with class II enhancers
Source: Sci Rep. 2021 Jan 12;11:510. doi: 10.1038/s41598-020-79980-x (PMC7804114; doi:10.1038/s41598-020-79980-x)
Supplement: Supplementary file 1 — Supplementary Information. [file 41598_2020_79980_MOESM1_ESM.pdf]

Supplementary Information

A class I odorant receptor enhancer shares a functional motif with class II enhancers

Tetsuo Iwata,[1,2¶] Satoshi Tomeoka,[3¶] and Junji Hirota[1, 3]

[1] Center for Biological Resources and Informatics, Tokyo Institute of Technology, Yokohama 226-8501, Japan

[2] Biomaterial Analysis Division, Technical Department, Tokyo Institute of Technology, Yokohama 226-8501, Japan

[3] Department of Life Science and Technology, Graduate School of Life Science and Technology, Tokyo Institute of Technology, Yokohama 226-8501, Japan

[¶]These authors contributed equally to this work.

Correspondence and requests for materials should be addressed to J.H. (email: jhirota@bio.titech.ac.jp)

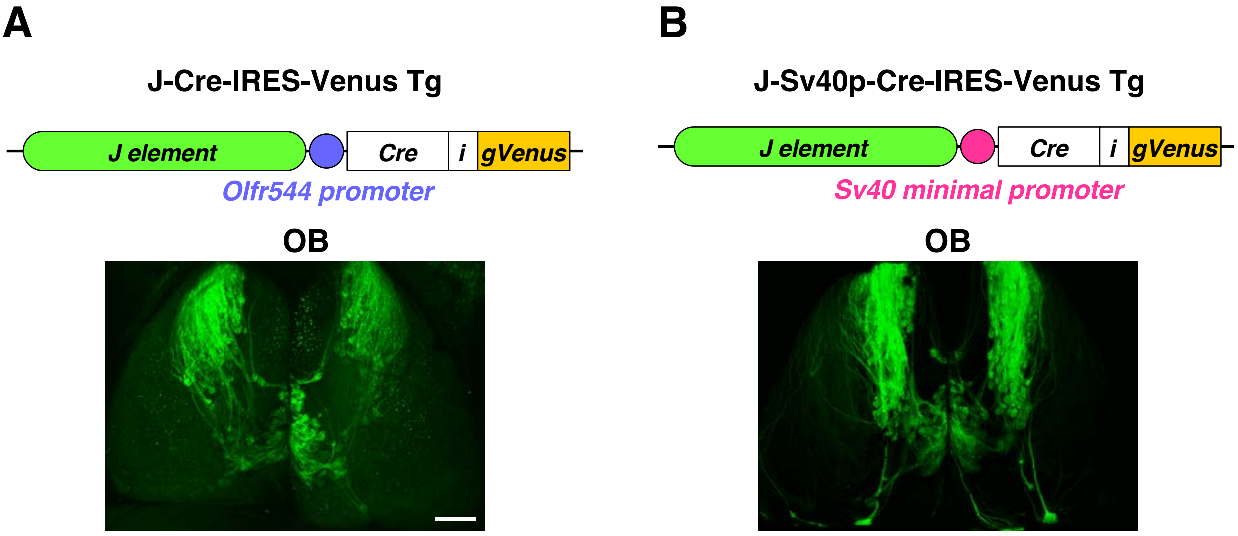

Supplementary Figure 1. Class I OSN-specific transcriptional activation of the J element does not depend on the promoter sequences. The Olfr544 promoter region itself could not activate reporter gene expression [13,26]. To confirm this, we replaced the Olfr544 promoter region of the J-Cre-IRES-gapVenus transgene （**A**) with the SV40 minimal promoter to construct the J-SV40p-Cre-IRES-gapVenus transgene (**B**). Both transgenic mice replicate the class I OSN-specific expression patterns. The scale bar is 500 µm.
